# Successful Pacing Profiles of Olympic Men and Women 3,000 m Steeplechasers

**DOI:** 10.3389/fspor.2020.00021

**Published:** 2020-03-11

**Authors:** Brian Hanley, Emily L. Williams

**Affiliations:** Carnegie School of Sport, Leeds Beckett University, Leeds, United Kingdom

**Keywords:** coaching, elite-standard athletes, endurance, race tactics, track and field

## Abstract

The presence of barriers in the steeplechase increases energy cost and makes successful pacing more difficult. This was the first study to analyze pacing profiles of successful (qualifiers for the final/Top 8 finalists) and unsuccessful (non-qualifiers/non-Top 8 finalists) Olympic steeplechasers across heats and finals, and to analyze differences between race sections (e.g., water jump vs. home straight). Finishing and section splits were collected for 77 men and 84 women competing at the 2008 and 2016 Olympic Games. Competitors were divided into groups based on finishing position (in both rounds analyzed). After a quick opening 228 m (no barriers), men who qualified for the final or finished in the Top 8 in the final had even paces for the first half with successive increases in speed in the last three laps; unsuccessful pacing profiles were more even. Successful women had mostly even paces for the whole race, and less successful athletes slowed after Lap 2. Women started the race relatively quicker than men, resulting in slower second half speeds. The best men completed most race sections at the same speed, but less successful men were slower during the water jump section, suggesting less technically proficiency. Similarly, women were slower during this section, possibly because its landing dimensions are the same as for men and have a greater effect on running speed. Coaches should note the different pacing profiles adopted by successful men and women steeplechasers, and the importance of technical hurdling skills at the water jump.

## Introduction

The 3,000 m steeplechase is an endurance event within track and field athletics, where competitors face the added difficulty of negotiating 35 rigid barriers, seven of which are water jumps. Each barrier is 0.914 m high in men's races and 0.762 m high in women's races (all other barrier dimensions are the same, including those of the water jump) (IAAF, 2017). The technique used to clear the barriers differs from that used in 400 m hurdling, even though the barriers are the same height (Chortiatinos et al., 2010). There are several reasons for this difference from 400 m hurdling technique: first, as the race is not run in lanes, the steeplechaser must hurdle in a crowd (Chortiatinos et al., 2010) and therefore needs to take extra care not to trip or misjudge the barrier; second, the gap between hurdles (of ~80 m) means that athletes do not have set, prepared stride patterns between them (Martin and Coe, 1997); third, the barriers are much sturdier and designed not to topple even from relatively large impacts (IAAF, 2017); and fourth, steeplechasers run at slower speeds than 400 m hurdlers. The barriers thus add an extra element of pacing skill required by athletes who must also take possible fatigue and tactical planning into consideration. In addition, the water jump section of each lap might be slower than other sections given the decelerating effect of the water pit and the tendency of most world-class athletes to step on the water jump barrier rather than hurdle it (Hanley et al., 2020). Despite its status as an Olympic event, the pacing profiles adopted by world-class steeplechasers have not previously been studied, and thus novel research with regard to both the overall race performance and the variation between different sections of the race (i.e., the home straight, back straight, first bend, and the water jump) will be beneficial to coaches when planning training regimens.

In distance running, managing one's physiological and psychological efforts is important in reaching the finish in the fastest possible time (Brick et al., 2016). Even-paced racing has a lower energy cost than racing with regular bursts of acceleration and deceleration (Padilla et al., 2000; Noorbergen et al., 2016); however, steeplechasers cannot avoid changes in speed to the same extent as other distance runners and the greater energy costs involved might affect the pacing profiles adopted. That said, previous research on pacing profiles shows that many world-class distance runners do not adopt an even pace in long-distance championships races (Hettinga et al., 2019), and one reason is that competitors tend to follow the pace set by others (Konings and Hettinga, 2018). It is possible that steeplechasers' pacing profiles are similarly affected by such collective behavior, even with the added difficulty of negotiating barriers. However, although previous studies on pacing in distance running can be used to examine successful approaches used by endurance athletes (e.g., Thiel et al., 2012), no previous research has examined the differences between successful and unsuccessful steeplechasers within championship racing, even though such information could be very valuable to steeplechase coaches when planning race strategies and the prior training required. In this study, success in the heats was considered qualifying for the final (in contrast to unsuccessful athletes who did not qualify), whereas in the final those athletes finishing in the Top 8 were considered successful.

The effort required to clear the barriers unsurprisingly results in greater energy costs (and slower finishing times) compared with running without them (Earl et al., 2015); the top 10 all-time best times for the men's steeplechase are ~30 s slower than those for the 3,000 m (non-championship) flat race, whereas the equivalent women's steeplechase times are ~45 s slower (IAAF, 2019). Men's steeplechase races first appeared at the Olympic Games in 1900 (Mallon, 1998) but a women's event was not held until 2008 (having first appeared at the IAAF World Championships in 2005) (IAAF, 2018). Given the differences in barrier heights between men's and women's events, it is possible that there are also technical effects on sex-based differences in pacing profiles that require specific approaches to training and that are of great importance to coaches. Indeed, previous research on sex-based differences in pacing has found that women are more conservative in the opening stages (Filipas et al., 2018) and overall are considered better pacers than men as they adopt more even paces when racing (March et al., 2011; Deaner et al., 2015). However, previous research on 1,500 and 5,000 m championship racing found that the best athletes had more varied paces [measured using coefficient of variation (CV)] than slower competitors (Hettinga et al., 2019), and running more even paces might therefore be disadvantageous. The aims of this study were to analyze successful and unsuccessful pacing profiles in Olympic steeplechasers using high-resolution, official electronic split times within heats and finals, to compare pacing profiles between sexes, and to analyze any differences between sections of the race. It was hypothesized that successful steeplechasers would race with less even paces than unsuccessful athletes, that successful women would have more even pacing than successful men, and that the water jump section would be completed slower than the home straight, back straight and first bend sections.

## Materials and Methods

### Research Approval

The protocol (application no. 51557) was approved by the Carnegie School of Sport Research Ethics Committee with the requirement for informed consent waived as the study analyzed publicly available data only. The study was conducted in accordance with the recognized ethical standards of the Declaration of Helsinki.

### Participants

Official electronic finishing and split times were obtained from results documents (IAAF, 2009; Almeida, 2016) for competitors in the men's and women's 3,000 m steeplechase races (heats and finals) at the Olympic Games in 2008 and 2016 (the only global championships with split times available). A total of 77 men's performances and 84 women's performances were analyzed across both championships. In total, the men represented 34 different nations and the women represented 38 different nations; including the six athletes who competed in both analyzed championships, the best represented nations (men/women) were the USA (6/6), Kenya (5/6), Ethiopia (5/4), Morocco (5/3), Spain (5/2), Canada (3/3), and Turkey (2/4). The performances of three men and three women considered very slow were omitted based on being highlighted as outliers using SPSS Statistics 24 (IBM SPSS, Inc., Chicago, IL), where an outlier was more than 1.5 times the interquartile range (IQR) from the median of the scores (Filipas et al., 2018). Athletes who did not finish in their heat or final (four men and five women) were not analyzed in those rounds. Because of faults in the timing system, the total complement of split times was not available for 18 women in the heats in 2008 and these athletes have been excluded from any analysis of their performances in the heats; the performances of those who qualified for the final were analyzed for that round only.

### Data Analysis

The study was designed as observational research in describing pacing profiles. Race split times were obtained for each section of the race (approximately one quarter of each lap) (IAAF, 2009; Almeida, 2016). Because the steeplechase requires a different track layout from a standard 400-m track to accommodate the water jump, each section was not exactly 100 m. The water jump in both championships analyzed was placed on the inside of the second bend, making this section 96 m long (which also meant that each race began 28 m before the normal 3,000 m start line). No barriers are crossed during the early part of the race (IAAF, 2017), so in this study the first 1.5 laps (approximately) have been described as comprising section 1 (228 m, no barriers), the first bend (100 m, one barrier), the back straight (100 m, two barriers), the water jump (96 m, one water jump), and the home straight (100 m, one barrier). With this 624 m completed, the latter four sections described above were then run a further six times to complete the full 3,000 m distance. To allow comparisons between sections of different lengths, mean speed was calculated for each section using the split times available.

Competitors were divided into groups based on finishing position (in the heats and finals), with men and women analyzed separately for this part of the study. There were two groups analyzed in the final: those who finished in the top eight (“Top 8”: 16 men; 16 women), and those who finished outside the top eight (“Non-Top 8”: 12 men; 14 women). Similarly, there were two groups analyzed in the heats: all those who qualified for the final (“Qualifiers”: 30 men; 24 women), and those who did not qualify (“Non-qualifiers”: 47 men; 53 women).

### Statistics

Results are reported as means ± one standard deviation (SD). One-way within-groups analysis of variance (ANOVA) was conducted on the mean speeds of each group with repeated contrast tests used to identify changes between successive race sections (Field, 2009). Greenhouse-Geisser corrections were used if Mauchly's test for sphericity was violated. In addition, independent *t*-tests were used to compare cumulative times between groups in each round (Field, 2009); groups were considered to have separated from one another when a difference was found between cumulative split times. To compare men's and women's pacing profiles, individuals' speeds for each section were expressed as a percentage of their mean speed for the whole race. These percentage data were arcsine transformed for the purposes of statistical analysis (Filipas et al., 2018) and compared using independent *t*-tests. Within-lap and between-lap variability was measured using CV and expressed as a percentage. Statistical significance was accepted as *p* < 0.05. Effect sizes for differences between successive sections, and between groups during each section, were calculated using Cohen's *d* (Cohen, 1988) and considered to be either trivial (*d* < 0.20), small (0.21–0.60), moderate (0.61–1.20), large (1.21–2.00), or very large (2.01–4.00) (Hopkins et al., 2009).

## Results

**Figure 1:**
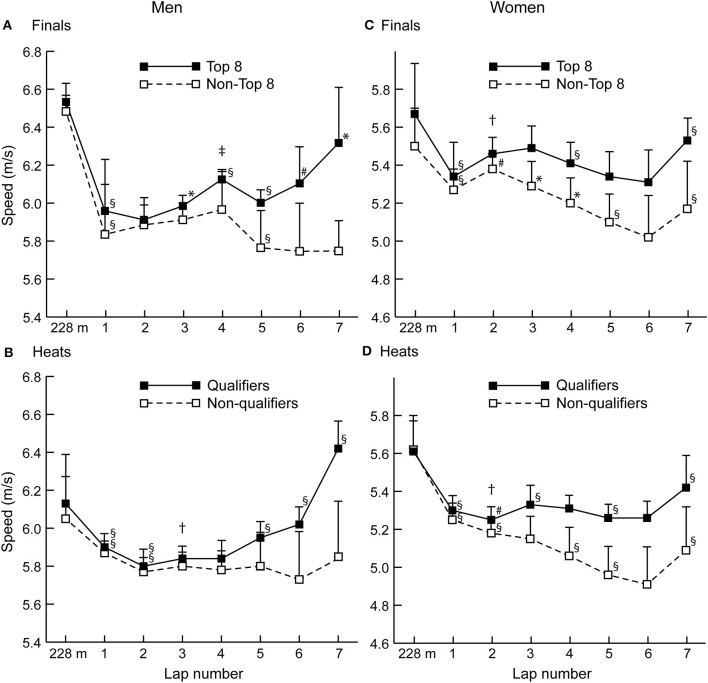
**(A–D)** The mean (+SD) section speed for each group of men and women athletes for finals and heats. Differences between successive sections with a moderate or larger effect size are shown as either ^§^*p* < 0.001, ^*^p < 0.01, or ^#^*p* < 0.05. Where separations between groups first occurred, these are indicated as either ^†^*p* < 0.01 or *p* < 0.05.

Mean lap speeds (including the first 228 m section) for each group of athletes are shown in Figures 1A–D, with annotations indicating where separation between groups (based on cumulative times) first occurred. In all figures (and the text below), differences between successive splits have been annotated when the effect size was moderate or larger only.

**Figure 2:**
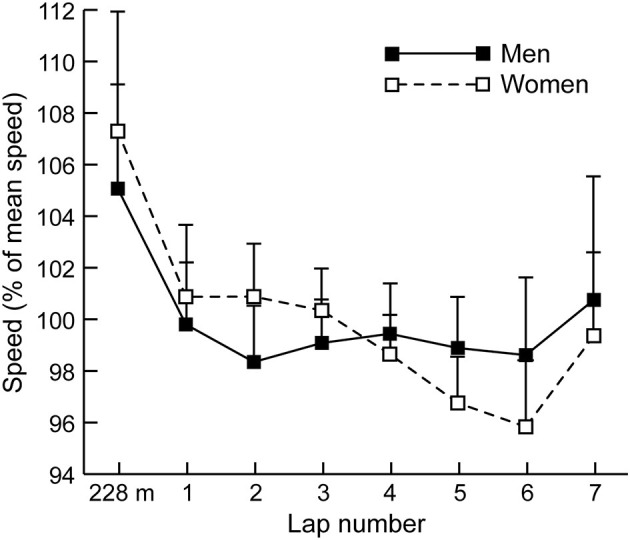
The mean (+SD) section speed expressed as a percentage of mean speed for all men and women.

With regard to all athletes who were included for analysis, the women's mean finishing time of 9:38.02 (±17.38) was 13.3% slower than the men's mean time (8:30.37 ± 12.72) (*p* < 0.001, *d* = 4.40). Figure 2 shows the mean lap speed percentages for all men and all women analyzed. Women were faster as a percentage of mean speed than the men during Lap 2 (*p* < 0.001, *d* = 1.07) and Lap 3 (*p* < 0.001, *d* = 0.66), but were relatively slower during Lap 5 (*p* < 0.001, *d* = 1.11) and Lap 6 (*p* < 0.001, *d* = 0.91). Across all men's races, 63% of the fastest speeds were run within the opening 228 m, whereas 21% were in the final home straight (2,900–3,000 m). Across all women's races, 62% of the fastest speeds were run within the opening 228 m, whereas 27% were in the final home straight.

Table 1 shows the mean speed for each group of men and women athletes for each of the four main sections of the race. Table 2 shows the mean CV for each group of men and women athletes for both within-lap and between-lap variability. The mean CV within laps for all men was 2.4% (±0.8), whereas it was 2.7% (±1.3) between laps; amongst all women, the mean CV within laps was 2.6% (±0.7), whereas it was 2.9% (±1.3) between laps.

**Table 1:** Mean (±SD) speeds (m/s) for each group of athletes for each section of the race.

	**Finals**		**Heats**
	**Top 8**	**Non-top 8**	**Non-qualifiers**
**MEN**			
First bend	6.02 (±0.10)^a^	5.88 (±0.10)	5.80 (±0.09)
Back straight	6.07 (±0.07)	5.85 (±0.14)	5.82 (±0.11)
Water jump	6.01 (±0.08)^a^	5.77 (±0.21)	5.70 (±0.14)^b^
Home straight	6.14 (±0.11)	5.89 (±0.09)	5.89 (±0.03)
**WOMEN**			
First bend	5.45 (±0.10)	5.23 (±0.11)	5.15 (±0.11)
Back straight	5.42 (±0.07)	5.23 (±0.14)	5.08 (±0.11)^a^
Water jump	5.29 (±0.08)^b^	5.09 (±0.12)^c^	4.95 (±0.13)^b^
Home straight	5.49 (±0.08)	5.28 (±0.13)	5.17 (±0.13)

a *Slower than the home straight (p < 0.01)*.

b *Slower than all other sections (p ≤ 0.001)*.

c *Slower than all other sections (p < 0.05)*.

**Table 2:** Mean (±SD) CV (%) both between and within laps for each group of men and women athletes.

	**Finals**		**Heats**
	**Top 8**	**Non-top 8**	**Non-qualifiers**
**MEN**			
Between laps	3.1 (±1.4)	3.2 (±1.6)	2.5 (±1.2)
Within laps	2.4 (±0.5)	2.7 (±1.5)	2.3 (±0.5)
**WOMEN**			
Between laps	2.5 (±0.7)	3.0 (±1.4)	3.0 (±1.4)
Within laps	2.4 (±0.3)	2.5 (±0.7)	2.7 (±0.7)

## Discussion

The aims of this study were to analyze successful and unsuccessful pacing profiles in Olympic steeplechasers within heats and finals, to compare pacing profiles between sexes, and to analyze any differences between sections of the race. It was hypothesized that successful steeplechasers would race with less even paces than unsuccessful athletes and, in this novel analysis of 3,000 m steeplechase pacing, the results indeed showed that most groups had parabolic shaped (U-shaped or reverse-J) pacing profiles (Abbiss and Laursen, 2008), with a quick opening section followed by a slower pace once the athletes started crossing the barriers, and a fast finish during the final stages of the race typical of racing with an endspurt finish (Renfree et al., 2012). In the men's event, the successful athletes mostly increased speed in the second half of the race, whereas the Non-qualifiers and Non-Top 8 ran very evenly paced races overall (with only one decrease in running speed found after Lap 2). The Non-Top 8 were unable to achieve similar increases in speed as they did during qualifying from the heats, suggesting that they might have been more affected by fatigue than the Top 8, or that reaching the final was the limit of their competitive capabilities. Although the frequently given advice that even paced racing is optimal for performance and is appropriate for achieving one's best time (Abbiss and Laursen, 2008), it was not shown to be the case in terms of achieving high finishing positions in men's steeplechase racing, and coaching practices for championship racing (which can differ from running to achieve a fast time) should reflect the need for increasingly fast laps toward the end of the race, as occurs in the similar track events of the 1,500 and 5,000 m (Hettinga et al., 2019).

Successful women's racing had a slightly different pacing profile, in that the Non-qualifiers and Non-Top 8 did not have even paced profiles (the Non-Top 8 slowed in each successive lap of the final from Lap 2 to Lap 5), whereas the more successful athletes had relatively even paced running with fast final laps; the Top 8 women only sped up once, on the final lap. This sex-based difference in pacing is highlighted by how women ran Laps 2 and 3 relatively faster than mean race pace compared with men (Figure 2), but ran Laps 5 and 6 slower. That women's mean times were ~13% slower than men's is typical of the sex-based differences found in other distance races (Cheuvront et al., 2005) but their less even pacing contrasts with previous findings on endurance races that women are more conservative in the opening stages (Filipas et al., 2018) or are considered better pacers than men (March et al., 2011; Deaner et al., 2015). Therefore, our hypothesis that successful women would have more even pacing than successful men was also rejected. Although there is no compulsory rationale for women to pace the same as men (or vice versa), an initial focus on adopting a slower pace in the first half of the race might be more physiologically beneficial for women if it means achieving a more even pace (Noorbergen et al., 2016). Training men and women with identical approaches should also be undertaken with caution given men's greater strength and statures; however, it should be noted that world-class women steeplechasers are taller relative to barrier height than men (Hanley et al., 2020).

Adopting the optimal performance-based pacing profile is not easy in a competitive environment where rewards are based on finishing position, as competitors tend to follow the pace set by others (Konings and Hettinga, 2018). Although this did occur in these steeplechase events, the separation between successful and unsuccessful athletes occurred relatively early in each case (as early as about 1,000 m in both women's finals and heats), and earlier than in other distance events such as the 5,000 m (Filipas et al., 2018; Hettinga et al., 2019). This early separation between groups might show that technically better athletes were able to quickly pull away from less skilled competitors. Men produced a faster start in the finals than in heats, possibly because of the higher standard of opponent, and the importance of the final could encourage a feeling of needing to keep in contact with the pack (Renfree and St Clair Gibson, 2013), which explains the slightly later separation of successful and unsuccessful men. The earlier separation in women's races could furthermore be an indication that the difference between technically competent and less skilled steeplechasers was even more pronounced, and would not be unexpected given the analyzed Olympic Games were the first and third appearances of the event for women at those championships. As with training for optimal competitive pacing in the steeplechase, which was overall less variable (Table 2) than in other middle- and long-distance races (Thiel et al., 2012), technical training for both men and women is crucial to avoid losing more time over the barriers than one's rivals.

Our hypothesis that the water jump section would be completed slower than the home straight, back straight and first bend sections was accepted as the comparisons between sections showed that the water jump was the slowest section for all groups of women. It was also slower than all other sections for the non-qualifying men in the heats, and slower than the home straight for the Top 8 finishers in the men's final. In the instance of the Top 8 men in the final, this might have indicated more that the home straight was particularly fast (it was the fastest section measured in any group), rather than that the water jump was slower than normal for these world-class men, especially as the first bend and back straight were not faster. The suggestion above that the Non-qualifiers were not as technically adept as the finalists in the men's event, and partly led to them being dropped from the lead pace quite early, is supported by the larger effect the water jump section had on their running speeds. Nearly all steeplechasers place their foot on the water jump barrier to push off it rather than hurdling it (Hanley et al., 2020), so it is not surprising that this particular section of each lap is slower, but the finding that all women's groups were slowest during the water jump section could also indicate that they were more affected by this barrier than men were. Indeed, the greatest difference between the sexes when matched by group was found during the water jump section (≥0.68 m/s) as previously identified in national-standard steeplechasers (Hunter et al., 2008). This might be because the dimensions of the water pit are the same for women as for men in terms of depth and width, even though the barrier height is lower. Furthermore, even some world-class women steeplechasers have poor water jump clearance techniques (Hanley et al., 2020) and landing from the jump is a key skill to develop alongside normal barrier clearance. Although the back straight was slower than the home straight for the non-qualifying women, there were no differences for other groups. This was despite two barriers being placed on this section, and shows that its placement very close to the end of the first bend means that both sections might be affected, with athletes decelerating at the end of the first bend to accommodate it (Kipp et al., 2017).

The main strength of this novel research is that the data have high ecological validity as the race splits were obtained from athletes competing in two Olympic Games. The results therefore reflect what occurs in real racing, where finishing position (whether in qualifying for the final or racing in the final itself) is more important than time achieved, and shows athletes and coaches what pacing strategies are adopted by the world's best steeplechasers. Thus, in terms of practical applications, coaches should note that steeplechasers need to train to be able to maintain a fast, but not excessive early pace, that allows them to keep up with the leaders and then increase running speed in the last, decisive laps. Being able to achieve these paces means ensuring the development of efficient barrier clearances in training, particularly at the water jump that was shown to affect slower athletes more. In terms of study limitations, since the steeplechase does not have a standard 400-m lap, official timing of each section is rare, and high-resolution split data were available for two major championships only; more measurements of split times will be invaluable in future studies of steeplechase pacing. As is typical of split data from Olympic Games athletics events used in pacing research (e.g., Thiel et al., 2012; Hettinga et al., 2019), data were available for each quarter-lap only and more precise measurements of pacing were not available. Another weakness of this study was that the data for several athletes was not available because of timing faults, and it should be noted that any study of pacing profiles using split data is not able to examine directly the tactical decisions that athletes took, or the reasons for them. Furthermore, participant data such as height, mass and training status were unavailable. However, the benefit of using these split data is that they reflect what actually occurred in racing, and future studies could examine whether women's pacing in the steeplechase continues to differ from men's or changes as they experience more championship racing opportunities, and empirically analyze what biomechanical factors reduce clearance time over the barriers.

## Conclusions

This study was the first to examine the pacing profiles of world-class steeplechasers, including the differences between successful and unsuccessful athletes, and sex-based differences. A typical parabolic pacing profile was seen for most groups, with faster opening and finishing sections. Successful athletes had a quicker endspurt than their unsuccessful counterparts in both heats and finals. For coaches of aspiring and successful athletes, assisting steeplechasers in their training regimens for elite-standard competition requires knowledge of these typical paces used and the changes that are likely to occur. When comparing the heats and finals, differences were seen in the separation distance of men, in which the possible importance of the final meant the Non-Top 8 were willing to stay with the pack for longer before reducing pace. Differences in technical ability in terms of crossing the barriers, and in particular the water jump, might have caused the early separation of the successful and unsuccessful athletes. In contrast with other distance running events, women had relatively quicker paces in the opening stages than men and a less even pacing profile overall, and stand to gain more from improvements in pacing and technical skills. Key points for coaches of elite-standard steeplechasers to note are that successful men need to be able to increase speed considerably in the second half of the race (and over successive laps), that women athletes should adopt a more conservative pace in the early stages of the race, and that athletes of both sexes need to develop technical proficiency over the barriers to reduce their effects on maintaining speed. Differences in relative strength and anthropometrics (e.g., height) between the sexes are important to consider when defining training strategies and in talent identification.

## Data Availability Statement

All datasets generated for this study are included in the article/Supplementary material.

## Ethics Statement

The studies involving human participants were reviewed and approved by Carnegie School of Sport Research Ethics Advisory Group. Written informed consent for participation was not required for this study in accordance with the national legislation and the institutional requirements.

## Author Contributions

BH and EW conceptualized and designed the study and wrote the manuscript. BH conducted the data collection and analyses and created tables. Both authors read and approved the final manuscript.

### Conflict of Interest

The authors declare that the research was conducted in the absence of any commercial or financial relationships that could be construed as a potential conflict of interest.

## References

[B1] Abbiss C. R. Laursen P. B. (2008). Describing and understanding pacing strategies during athletic competition. Sports Med. 38, 239–252. 10.2165/00007256-200838030-00004 18278984

[B2] Almeida A. (2016). Rio 2016 Results Book: Athletics. Rio de Janeiro: Rio 2016 Organising Committee.

[B3] Brick N. E. Campbell M. J. Metcalfe R. S. Mair J. L. Macintyre T. E. (2016). Altering pace control and pace regulation: attentional focus effects during running. Med. Sci. Sports Exerc. 48, 879–886. 10.1249/MSS.0000000000000843 26673128

[B4] Cheuvront S. N. Carter R. DeRuisseau K. C. Moffatt R. J. (2005). Running performance differences between men and women: an update. Sports Med. 35, 1017–1024. 10.2165/00007256-200535120-00002 16336006

[B5] Chortiatinos G. X. Panoutsakopoulos V. Kollias I. A. (2010). 3D biomechanical analysis of Galina-Samitova's steeplechase hurdling. New Stud. Athlet. 25, 81–93.

[B6] Cohen J. (1988). Statistical Power Analysis for the Behavioural Sciences, 2nd Edn. Hillsdale, NJ: Lawrence Erlbaum.

[B7] Deaner R. O. Carter R. E. Joyner M. J. Hunter S. K. (2015). Men are more likely than women to slow in the marathon. Med. Sci. Sports Exerc. 47, 607–616. 10.1249/MSS.0000000000000432 24983344 PMC4289124

[B8] Earl S. Hunter I. Mack G. W. Seeley M. (2015). The relationship between steeplechase hurdle economy, mechanics, and performance. J. Sport Health Sci. 4, 353–356. 10.1016/j.jshs.2015.03.009

[B9] Field A. P. (2009). Discovering Statistics Using SPSS, 4th Edn. London: Sage.

[B10] Filipas L. La Torre A. Hanley B. (2018). Pacing profiles of olympic and IAAF world championship long-distance runners. J. Strength Cond. Res. 10.1519/JSC.0000000000002873. [Epub ahead of print]. 30289868

[B11] Hanley B. Bissas A. Merlino S. (2020). Better water jump clearances were differentiated by longer landing distances in the 2017 IAAF World Championship 3000 m steeplechase finals. J. Sports Sci. 38, 380–385. 10.1080/02640414.2019.1698091 31774365

[B12] Hettinga F. J. Edwards A. M. Hanley B. (2019). The science behind competition and winning in athletics: using world-level competition data to explore pacing and tactics. Front. Sports Act. Living 1:11 10.3389/fspor.2019.00011 PMC7739697 33344935

[B13] Hopkins W. G. Marshall S. W. Batterham A. M. Hanin J. (2009). Progressive statistics for studies in sports medicine and exercise science. Med. Sci. Sports Exerc. 41, 3–12. 10.1249/MSS.0b013e31818cb278 19092709

[B14] Hunter I. Lindsay B. K. Andersen K. R. (2008). Gender differences and biomechanics in the 3000m steeplechase water jump. J. Sports Sci. Med. 7, 218–222. Retrieved from: http://www.jssm.org/abstresearchajssm-07-218.xml 24149452 PMC3761453

[B15] IAAF (2009). Beijing Distance Races Analysed at 100m Intervals. Available online at: https://www.iaaf.org/news/news/beijing-distance-races-analysed-at-100m-inter (accessed August 21, 2019).

[B16] IAAF (2017). Competition Rules 2018–2019. Monte Carlo: IAAF.

[B17] IAAF (2018). Competition Archive. Available online at: iaaf.org http://www.iaaf.org/results?&subcats=WCH,OLY (accessed August 21, 2019).

[B18] IAAF (2019). Records & Lists. Available online at: iaaf.org http://www.iaaf.org/records/toplists/road-running/marathon/outdoor/women/senior (accessed August 21, 2019)

[B19] Kipp S. Taboga P. Kram R. (2017). Ground reaction forces during steeplechase hurdling and water jumps. Sports Biomech. 16, 152–165. 10.1080/14763141.2016.1212917 27592823

[B20] Konings M. J. Hettinga F. J. (2018). The impact of different competitive environments on pacing and performance. Int. J. Sports Physiol. Perform. 13, 701–708. 10.1123/ijspp.2017-0407 29035590

[B21] Mallon B. (1998). The 1900 Olympic Games: Results for All Competitors in All Events, With Commentary. Jefferson, NC: McFarland & Company, Inc.

[B22] March D. S. Vanderburgh P. M. Titlebaum P. J. Hoops M. L. (2011). Age, sex, and finish time as determinants of pacing in the marathon. J. Strength Cond. Res. 25, 386–391. 10.1519/JSC.0b013e3181bffd0f 20224445

[B23] Martin D. E. Coe P. N. (1997). Better Training for Distance Runners, 2nd Edn. Champaign, IL: Human Kinetics.

[B24] Noorbergen O. S. Konings M. J. Micklewright D. Elferink-Gemser M. T. Hettinga F. J. (2016). Pacing behavior and tactical positioning in 500- and 1000-m short-track speed skating. Int. J. Sports Physiol. Perform. 11, 742–748. 10.1123/ijspp.2015-0384 26641204

[B25] Padilla S. Mujika I. Angulo F. Goiriena J. J. (2000). Scientific approach to the 1-h cycling world record: a case study. J. Appl. Physiol. 89, 1522–1527. 10.1152/jappl.2000.89.4.1522 11007591

[B26] Renfree A. St Clair Gibson A. (2013). Influence of different performance levels on pacing strategy during the women's World Championship marathon race. Int. J. Sports Physiol. Perform. 8, 279–285. 10.1123/ijspp.8.3.279 23006811

[B27] Renfree A. West J. Corbett M. Rhoden C. St Clair Gibson A. (2012). Complex interplay between determinants of pacing and performance during 20-km cycle time trials. Int. J. Sports Physiol. Perform. 7, 121–129. 10.1123/ijspp.7.2.121 22173069

[B28] Thiel C. Foster C. Banzer W. de Koning J. (2012). Pacing in olympic track races: competitive tactics versus best performance strategy. J. Sports Sci. 30, 1107–1115. 10.1080/02640414.2012.701759 22738897

